# Single-Cell Transcriptome Profiling Unravels Distinct Peripheral Blood Immune Cell Signatures of RRMS and MOG Antibody-Associated Disease

**DOI:** 10.3389/fneur.2021.807646

**Published:** 2022-01-14

**Authors:** Ju Liu, Xiaoyan Yang, Jiali Pan, Zhihua Wei, Peidong Liu, Min Chen, Hongbo Liu

**Affiliations:** ^1^Department of Neurology, The First Affiliated Hospital of Zhengzhou University, Zhengzhou, China; ^2^Department of Neurosurgery, The First Affiliated Hospital of Zhengzhou University, Zhengzhou, China

**Keywords:** RRMS, MOGAD, peripheral blood, single-cell RNA sequencing, biomarker

## Abstract

Relapsing-remitting multiple sclerosis (RRMS) and myelin oligodendrocyte glycoprotein (MOG) antibody-associated disease (MOGAD) are inflammatory demyelinating diseases of the central nervous system (CNS). Due to the shared clinical manifestations, detection of disease-specific serum antibody of the two diseases is currently considered as the gold standard for the diagnosis; however, the serum antibody levels are unpredictable during different stages of the two diseases. Herein, peripheral blood single-cell transcriptome was used to unveil distinct immune cell signatures of the two diseases, with the aim to provide predictive discrimination. Single-cell RNA sequencing (scRNA-seq) was conducted on the peripheral blood from three subjects, i.e., one patient with RRMS, one patient with MOGAD, and one patient with healthy control. The results showed that the CD19^+^ CXCR4^+^ naive B cell subsets were significantly expanded in both RRMS and MOGAD, which was verified by flow cytometry. More importantly, RRMS single-cell transcriptomic was characterized by increased naive CD8^+^ T cells and cytotoxic memory-like Natural Killer (NK) cells, together with decreased inflammatory monocytes, whereas MOGAD exhibited increased inflammatory monocytes and cytotoxic CD8 effector T cells, coupled with decreased plasma cells and memory B cells. Collectively, our findings indicate that the two diseases exhibit distinct immune cell signatures, which allows for highly predictive discrimination of the two diseases and paves a novel avenue for diagnosis and therapy of neuroinflammatory diseases.

## Introduction

Multiple sclerosis (MS) is a common chronic inflammatory demyelinating disease of the central nervous system (CNS) and the leading cause of neurologic disability in young adults (1). Globally, ~30–300 per 100,000 adults are affected with MS, leading to a substantial economic burden on healthcare systems and societies (2, 3). MS repertoire manifests pathological characteristics of inflammation, demyelination, and axonal damage in the CNS. Among MS, relapsing-remitting MS (RRMS) is the most common type, accounting for nearly 85% of the initial diagnoses (4–6). Myelin oligodendrocyte glycoprotein (MOG) antibody-associated disease (MOGAD) is a newly classified inflammatory demyelinating disease of CNS that shares clinical manifestations with RRMS (7–10). Hence, the detection of disease-specific serum antibody is currently regarded as the gold standard for their diagnosis, however, the serum antibody levels are unpredictable at different stages of the two diseases (11). Therefore, discovering antibody-independent biomarkers is needed.

Technological advances in single-cell RNA sequencing (scRNA-seq) have improved the understanding of the immunopathology of numerous autoimmune diseases by identifying diagnostic biomarkers (12–14). Hong et al. studied the immune cells of peripheral blood mononuclear cells (PBMCs) of patients with Primary Sjögren's syndrome by using scRNA-seq and identified some disease-specific immune cell subsets (15). Likewise, Ramesh et al. used scRNA-seq to characterize the CNS-specific B cell phenotypes in MS with paired immune repertoires and further confirmed the pathogenic role of B cells in the CNS of patients with MS (16). Schafflick et al. used single-cell transcriptomics to describe the leukocytes of cerebrospinal fluid (CSF) and identified the specific composition and transcriptome of CSF leukocytes (17). In another study, scRNA-seq was conducted on the CSF of patients with RRMS and MOGAD, and the shared myeloid populations were identified (18). However, scRNA-seq has been rarely used to decipher peripheral blood signatures of patients with RRMS or MOGAD.

In this study, peripheral blood single-cell transcriptome was used to identify immune cell signatures of RRMS and MOGAD. Our findings unveil distinct signatures of peripheral blood immune cells of patients with RRMS or MOGAD and provide a reference for diagnostic and therapeutic intervention in neuroinflammatory diseases.

## Materials and Methods

### Subjects

The study was reviewed and approved by the Ethics Committee of the First Affiliated Hospital of Zhengzhou University (2021-KY-0588-002). Before the study, written informed consent was signed by each participant. Patients with RRMS or MOGAD and healthy controls (HCs) were recruited in this study (Table 1). The patients with RRMS and MOGAD were confirmed according to the McDonald criteria (19) and MOGAD diagnostic criteria (20), respectively. The exclusion criteria were (1) prior treatment with the immunosuppressants (e.g., azathioprine, mycophenolate mofetil, and even corticosteroids) or disease-modifying therapies (e.g., teriflunomide, fingolimod, and siponimod); (2) coexisting autoimmune disorders (e.g., systemic lupus erythematosus or Sjogren's syndrome); (3) positive with other autoimmune antibodies (e.g., anti-N-methyl D-aspartate (NMDA) receptor antibodies); (4) acute or chronic infections (e.g., respiratory tract infections, hepatitis, or tuberculosis); (5) organ dysfunction; and (6) in stable phase or in remission. The peripheral blood of each participant was collected for subsequent analysis.

**Table 1:** Demographic and clinical features of subjects.

**Subject**	**Diagnosis**	**Sex**	**Age (years)**	**Race**	**Presentation at acute phase**	**MOG antibody titer (serum)**	**Cell counts/μl**	**Protein, mg/dl**	**OB**	**Analysis**
1	MOGAD	M	53	Han Chinese	Encephalitis and epileptic seizure	1:100	50	44	I	scRNA-seq
2	RRMS	M	37	Han Chinese	Numbness and weakness of limbs	Negative	5	23	II	scRNA-seq
3	HC	M	38	Han Chinese	NA	NA	NA	NA	NA	scRNA-seq
4	MOGAD	F	22	Han Chinese	Optic neuritis	1:10	9	13	I	Flow cytometry
5	MOGAD	M	20	Han Chinese	Encephalitis and seizure	1:1000	29	19	I	Flow cytometry
6	MOGAD	M	49	Han Chinese	ADEM	1:100	10	26	I	Flow cytometry
7	MOGAD	F	50	Han Chinese	Myelitis	1:320	36	43	I	Flow cytometry
8	MOGAD	F	33	Han Chinese	Encephalitis	1:32	32	41	I	Flow cytometry
9	RRMS	F	47	Han Chinese	Left limb weakness	Negative	10	40	I	Flow cytometry
10	RRMS	M	26	Han Chinese	Cerebellar ataxia	Negative	2	18	IV	Flow cytometry
11	RRMS	F	64	Han Chinese	lower-extremity weakness	Negative	4	38	II	Flow cytometry
12	RRMS	M	22	Han Chinese	Left lower-limb numbness and weakness	Negative	4	17	I	Flow cytometry
13	RRMS	F	21	Han Chinese	Diplopia and eye movement disorders	Negative	8	21	II	Flow cytometry
14	HC	M	49	Han Chinese	NA	NA	NA	NA	NA	Flow cytometry
15	HC	M	30	Han Chinese	NA	NA	NA	NA	NA	Flow cytometry
16	HC	F	20	Han Chinese	NA	NA	NA	NA	NA	Flow cytometry
17	HC	F	54	Han Chinese	NA	NA	NA	NA	NA	Flow cytometry
18	HC	F	52	Han Chinese	NA	NA	NA	NA	NA	Flow cytometry

*ADEM, acute disseminated encephalomyelitis; HC, healthy control; ON, optic neuritis; OB, oligoclonal band; MOGAD, myelin oligodendrocyte glycoprotein (MOG) antibody-associated disease; NA, not applicable; RRMS, relapsing-remitting multiple sclerosis; scRNA-seq, single-cell RNA sequencing*.

### Capturing and Sequencing of Single-Cell Data

As previously described (21), the PBMCs were isolated and then resuspended in prechilled phosphate-buffered saline. Trypan blue staining was used to confirm the cell viability. The single-cell suspension with more than 90% of cells was loaded onto 10 × Genomics Chromium Controller using Chromium Single Cell 3' Library and Gel Bead Kit v2 (10 × Genomics). The libraries were constructed according to the guidelines of the manufacturer. The libraries were purified by AMPure beads (Beckman Coulter, Krefeld, Germany) and then sequenced on an Illumina NovaSeq 6000 platform with 150 bp paired-end mode.

### Analysis of Single-Cell Sequence Data

Data analysis for single-cell sequencing was performed as previously described (22). Briefly, read files were extracted using the Cell Ranger pipeline v2.2.0 (10 × Genomics) and then aligned to the human GRCh38 genome to generate gene expression data for each cell. Double entries were filtered using Scrublet software (23), followed by exporting the filtered gene expression matrix data into Seurat software v4.0 (24) to perform subsequent analysis. The high-quality single-cell data were normalized using the LogNormalize function, and principal component analysis was carried out. The significant principal component (*p* <1^e−5^) was selected to perform cluster analysis. The single cells were clustered by t-Distributed Stochastic Neighbor Embedding (tSNE), and the clusters were classified based on established markers from the CellMarker database (25). Final single-cell data visualization and exploration were generated by tSNE (26). The sequenced data have been deposited into the National Center for Biotechnology Information (NCBI) BioProject database with accession number PRJNA776659.

### Flow Cytometry

Fifteen subjects, i.e., five RRMS, five MOGAD, and five HC, were recruited to conduct flow cytometry analysis (27). In brief, after removing erythrocytes using lysing solution (BD Biosciences, San Diego, CA, USA), the staining solution containing ghost dye (Tonbobio, Beijing, China) and human monoclonal specific antibody CD19 was used to stain the samples at 4°C for 30 min, and then the samples were permeated for 30 min at room temperature and then was stained with CXCR4 antibody for 30 min at room temperature. The re-suspended cells were run on a BD FACS Canto II flow cytometer (BD Biosciences, San Diego, CA, USA), and the cells were analyzed using FlowJo software (Tree Star, Ashland, OR, USA).

The antibodies used in this study to stain cells included Allophycocyanin (APC) anti-human CD19 antibody (clone SJ25C1; BioLegend, San Diego, CA, USA) 1:20, and PE anti-human CD184 (CXCR4) antibody (clone 12G5; BioLegend, San Diego, CA, USA) 1:20.

### Statistical Analyses

Statistical analysis was done using Graphpad Prism 9 software (GraphPad Software Inc, La Jolla, CA, USA). One-way ANOVA was used to analyze the difference among multiple groups. The data represent the mean ± SEM. A *p* < 0.05 was considered statistically significant.

## Results

### Single-Cell Transcriptomic of Peripheral Blood

**Figure 1:**
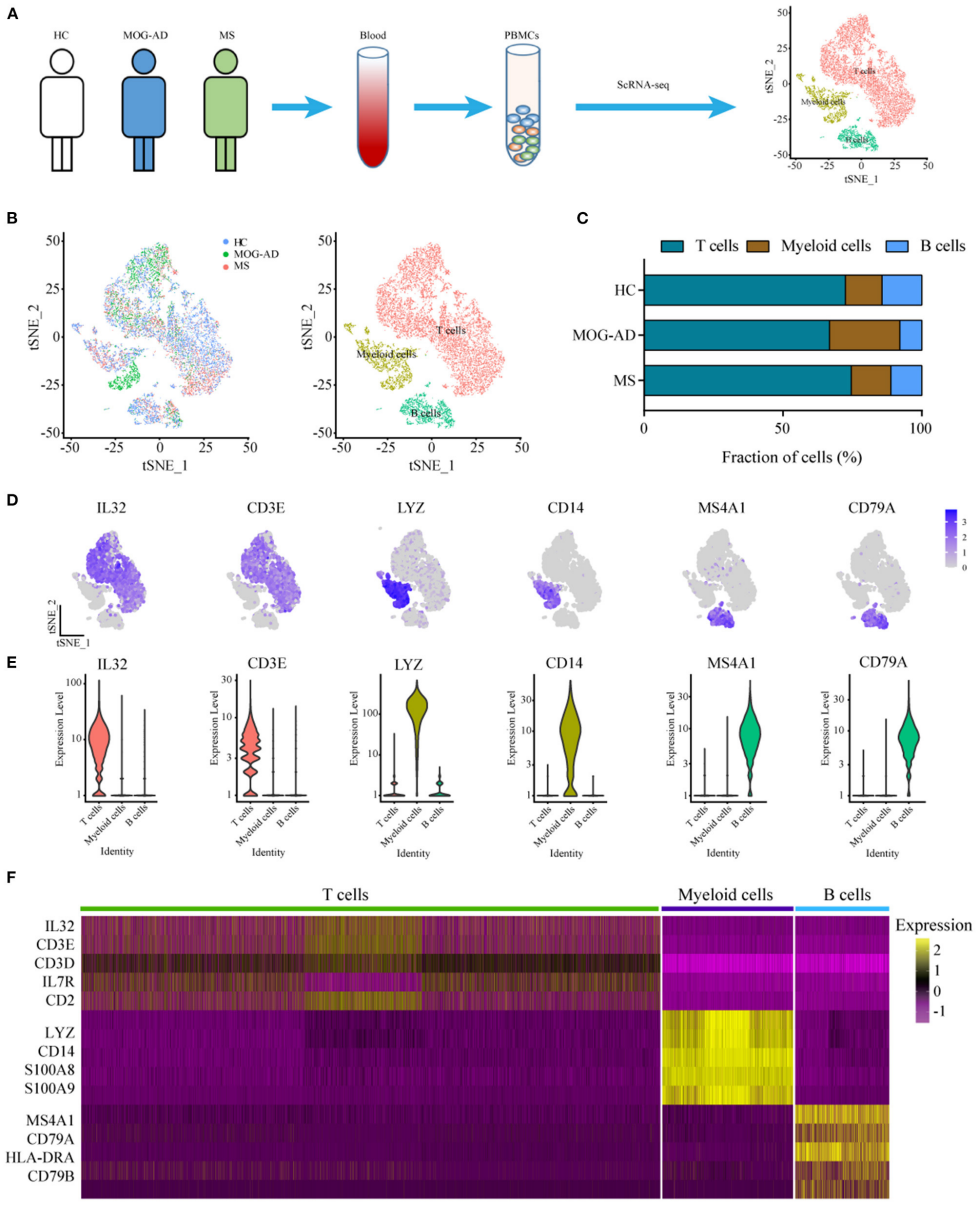
Single-cell transcriptional profiling of PBMCs from HC, RRMS, and MOGAD. **(A)** The experimental workflow for obtaining and analyzing PBMC between all three donor groups. **(B)** The t-Distributed Stochastic Neighbor Embedding (tSNE) was used to visualize cell populations generated by unsupervised cluster analysis of PBMC. **(C)** The percentage of each cell type in HC, MS, and MOGAD. **(D)** The expression level of the marker gene in each cell type was shown. **(E)** Violin illustration shows the expression of marker genes from different known cell types. **(F)** Heatmaps shows the expression of the up- and downregulated genes in T, B, and myeloid cells. HC, healthy controls; RRMS, relapsing-remitting multiple sclerosis; MOGAD, myelin oligodendrocyte glycoprotein (MOG) antibody-associated disease; PBMC, peripheral blood mononuclear cell.

To identify the characteristics of immune-cell subsets of peripheral blood of the patients with RRMS or MOGAD, scRNA-seq of PBMCs was performed (Figure 1A). A total of 18,016 cells from PBMCs (7,709 cells from HC, 3,969 cells from MOGAD, and 6,338 cells from MS) were isolated and sequenced. After removing the duplicate cells, low-quality, and empty droplets, 15,252 cells were finally collected and used in the subsequent analysis (Supplementary Figure 1 and Supplementary Table 1). Unsupervised clustering analysis identified three distinct immune cell clusters (Figure 1B and Supplementary Table 2). Cluster 1 (~72.95%) was identified as T cells based on the expression of marker genes IL32, CD3E, IL7R, CD3D, and CD2 (Figures 1C–F). Cluster 2 (~14.17%) was identified as B cells based on the expression of marker genes MS4A1, CD79A, HLA-DRA, and CD79B (Figures 1C–F). Cluster 3 (~12.87%) was classified as myeloid cells according to the expression of marker genes LYZ, CD14, S100A8, and S100A9 (Figures 1C–F). Additionally, a large set of other markers were also identified, such as GIMAP7, CD247, and LCK for T cells, ADAM28, VPREB3, and BANK1 for B cells, LST1, MNDA, FCN1, and SERPINA1 for myeloid cells (Supplementary Table 3). We focused on the characteristics of RRMS and MOGAD based on the three immune cell clusters in the above analysis.

### Characteristics of Myeloid Cell Subsets Between RRMS and MOGAD

**Figure 2:**
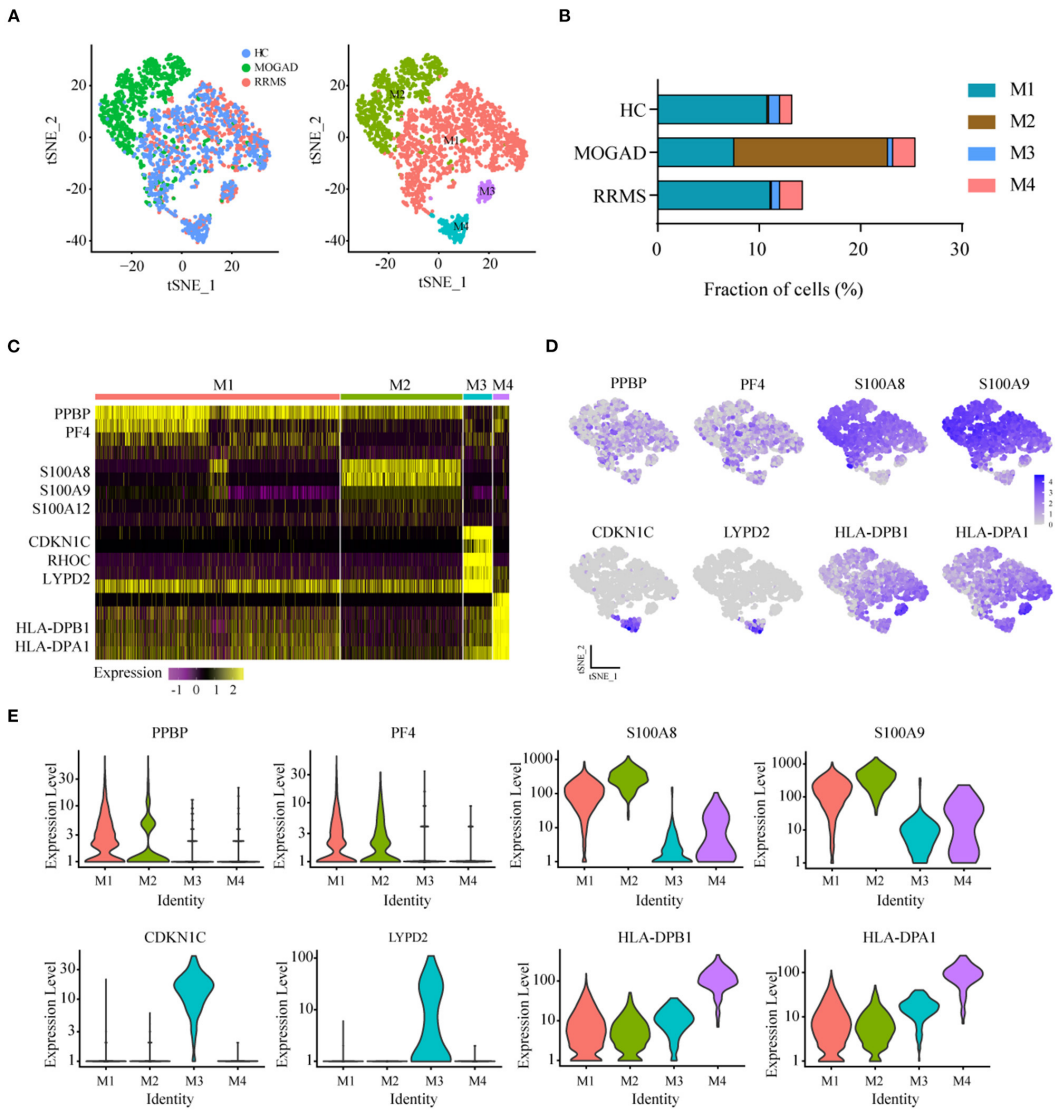
Identification and analysis of myeloid clusters in PBMC from HC, RRMS, and MOGAD. **(A)** tSNE visualization of myeloid cells from HC, RRMS, and MOGAD. **(B)** The percentage of cells for myeloid subsets in HC, RRMS, and MOGAD. **(C)** Heatmaps shows the expression of the up- and downregulated markers in myeloid subsets. **(D)** The expression level of marker gene in each myeloid cell type was shown. **(E)** Violin illustration showed the expression of the marker genes from different myeloid cell types. HC, healthy controls; RRMS, relapsing-remitting multiple sclerosis; MOGAD, myelin oligodendrocyte glycoprotein (MOG) antibody-associated disease; PBMC, peripheral blood mononuclear cell; tSNE, t-Distributed Stochastic Neighbor Embedding.

Further unsupervised clustering regarding the myeloid cells was performed to understand the changes of myeloid cell clusters in RRMS and MOGAD. The results showed that three monocyte subsets, classical (CD14^++^ CD16^−^), non-classical (CD14^+^ CD16^++^), and intermediate (CD14^++^ CD16^+^) monocytes, were identified based on the distinct markers, which were observed to be shared among all subjects (Figures 2A,B). Among the monocyte subsets, the CD14^++^ CD16^−^ cells were further subdivided into M1 and M2 based on the distinct markers where M1 cells expressed PPBP (CXXL7) and PF4 (CXCL4). In contrast, the M2 subset (inflammatory monocytes) expressed a high level of S100A8, S100A9, and S100A12 (Figures 2C–E, Supplementary Table 4). CD14^+^ CD16^++^ cells (M3) expressed markers CDKN1C, RHOC, and LYPD2, without S100A12 (Figures 2C–E and Supplementary Table 4). The intermediate monocytes (M4) expressed high levels of marker HLA-DPB1 and HLA-DPA1.

The percentage of M4 intermediate (CD14^++^ CD16^+^) monocytes was relatively higher in RRMS (2.13%) and MOGAD (1.98%) than in the HCs (1.11%) in the identified myeloid cell subsets. A comparison between RRMS and MOGAD revealed that M1 monocytes were reduced while M2 monocytes were increased in MOGAD (Figure 2B and Supplementary Table 2). The above-identified four subsets in myeloid cells showed that M4 intermediate (CD14^++^ CD16^+^) monocytes were expanded in RRMS and MOGAD, but M2 inflammatory monocytes were specifically enriched in MOGAD.

### Characteristics of T Cell Subsets Between RRMS and MOGAD

**Figure 3:**
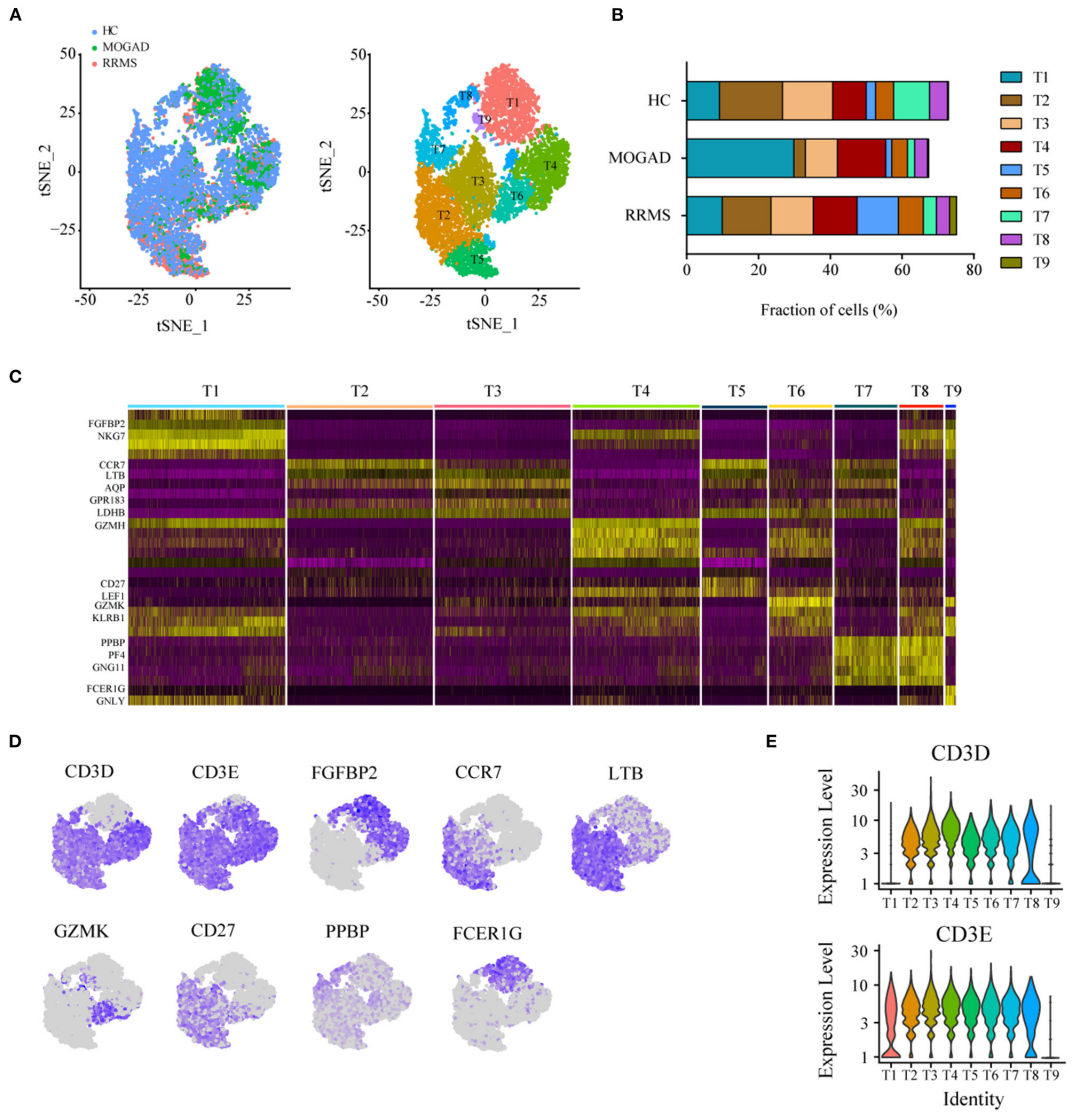
Analysis of T cell clusters from HC, RRMS, and MOGAD. **(A)** tSNE visualization of T cells from HC, RRMS, and MOGAD. **(B)** The percentage of cells for T cell subsets in HC, RRMS, and MOGAD. **(C)** Heatmaps shows the expression of the up- and downregulated markers in T cell subsets. **(D)** The expression level of marker genes in each T cell type was shown. **(E)** Violin illustration shows the expression of the marker genes from different T cell types. HC, healthy controls; RRMS, relapsing-remitting multiple sclerosis; MOGAD, myelin oligodendrocyte glycoprotein (MOG) antibody-associated disease; PBMC, peripheral blood mononuclear cell; tSNE, t-Distributed Stochastic Neighbor Embedding.

**Figure 4:**
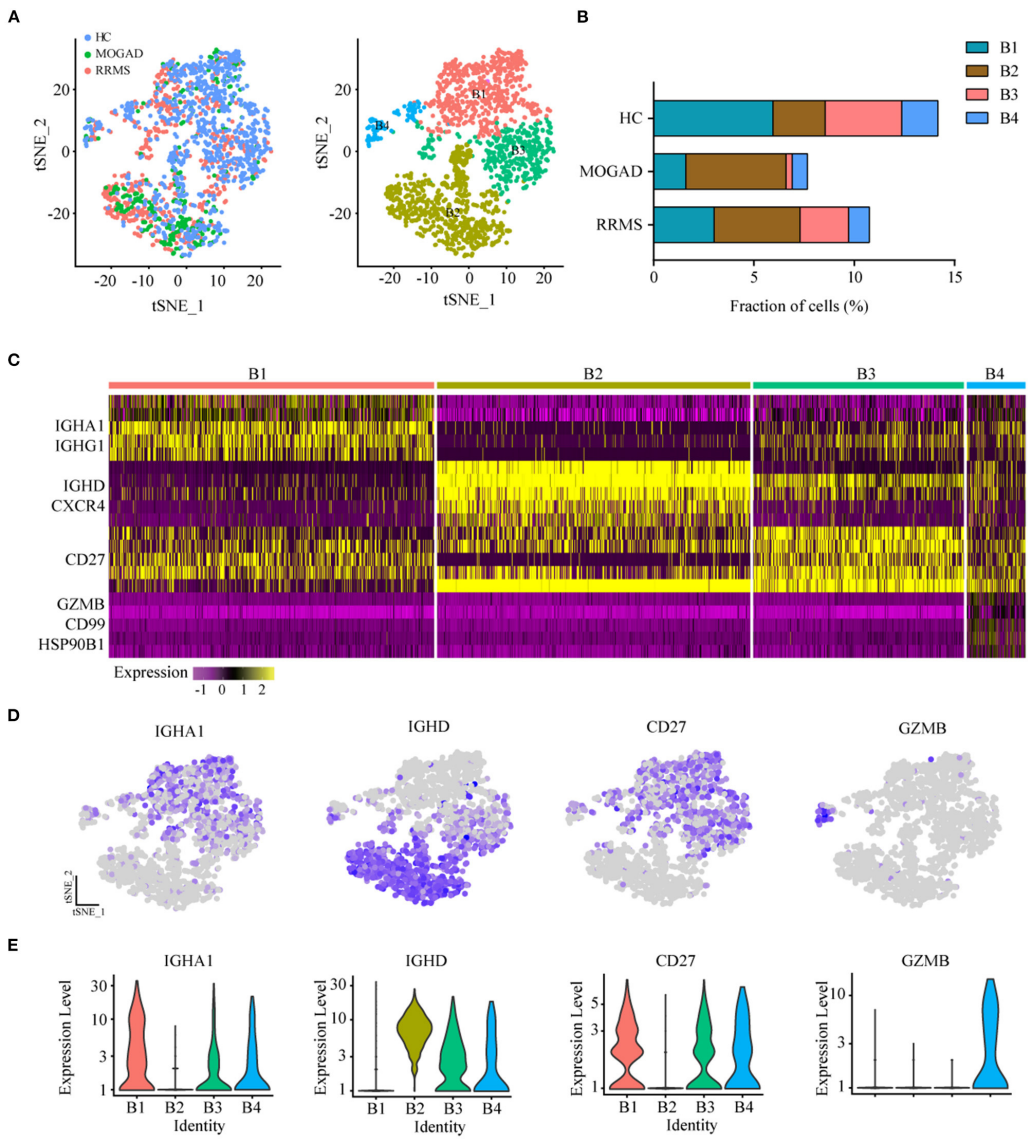
Analysis of B cell clusters from HC, RRMS, and MOGAD. **(A)** tSNE visualization of B cells from HC, RRMS, and MOGAD. **(B)** The percentage of cells for B cell subsets in HC, RRMS, and MOGAD. **(C)** Heatmaps shows the expression of the up- and downregulated markers in B cell subsets. **(D)** The expression level of marker gene in each B cell type was shown. **(E)** Violin illustration shows the expression of the marker genes from different B cell types. HC, healthy controls; RRMS, relapsing-remitting multiple sclerosis; MOGAD, myelin oligodendrocyte glycoprotein (MOG) antibody-associated disease. PBMC, peripheral blood mononuclear cell; tSNE, t-Distributed Stochastic Neighbor Embedding.

The unsupervised clustering of T cells was conducted to understand the differences in T cell clusters between RRMS and MOGAD. Nine sub-clusters (T1-T9) were identified from 10,610 T cells based on the specific markers (Figures 3A–D and Supplementary Table 5), and 8 clusters (T1–T8) expressed high levels of CD3D and CD3E (Figures 3D,E). Two distinct CD4^+^ T cell subsets were identified, such as naive-like CD4 T cells (T2) and T3. T2 was identified based on the expressed marker CCR7, and T3 was based on the expressed marker TNFSF3 (LTβ, an activated CD4 T-cell marker), AQP, GPR183, and LDHB (Supplementary Table 5). Six distinct CD8^+^ T cell clusters were identified, such as cytotoxic CD8 effector T cells (T1 and T4: expressed markers FGFBP2, NKG7, and GZMH), transitional CD8 effector T cells (T6: expressed markers GZMK and KLRB1), naive CD8^+^ T cells (T5: expressed markers CD27 and LEF1), and megakaryocyte-like cells (T7 and T8: expressed markers PPBP, PF4, and GNG11) (Figures 4A–D and Supplementary Table 5). In addition, we can discriminate cluster T9 from T cells based on the lower expression levels of CD3D and CD3E, indicating that cluster T9 may be Natural Killer (NK) cells. Further, the higher levels of FCER1G, GNLY, CD7, KLRF1, and KLRC2 in the T9 were also observed, implying that T9 can be classified as cytotoxic memory-like NK cells (Figures 3A–E and Supplementary Table 5).

Compared with HCs, the levels of cytotoxic CD8 effector T cells (T4) and cytotoxic memory-like NK cells (T9) were higher in both RRMS and MOGAD, whereas the fraction of megakaryocyte-like cells (T7) was relatively lower. A comparison between RRMS and MOGAD showed that the fraction of T1 subsets was increased while T5 was decreased in MOGAD (Figure 3B and Supplementary Table 2).

### Characteristics of B Cell Subsets in RRMS and MOGAD

The unsupervised clustering on B cells was performed to identify the characteristics of B cell subsets in RRMS and MOGAD. The results showed that four distinct B cell clusters, i.e., plasma (B1), naive B (B2), memory B (B3) cells, and plasmacytoid DCs (B4), were identified from 1,676 B cells based on the markers from the CellMarker database (Figures 4A–C and Supplementary Table 6). Among 4 B cell subsets (Figures 4C–E and Supplementary Table 6), the B1 subset expressed plasma cell markers IGHA1 and IGHG1, indicating the presence of plasma cells. B2 subset expressed high levels of IL10 (IL10RA) and naive B cell markers IGHD and CXCR4, showing the presence of naive B cells. The B3 cluster with high expression of CD27 was similar to memory B cell, and the B4 subset based on the high expression of GZMB, CD99, and HSP90B1 was similar to plasmacytoid dendritic cells (pDC)-like cells.

**Figure 5:**
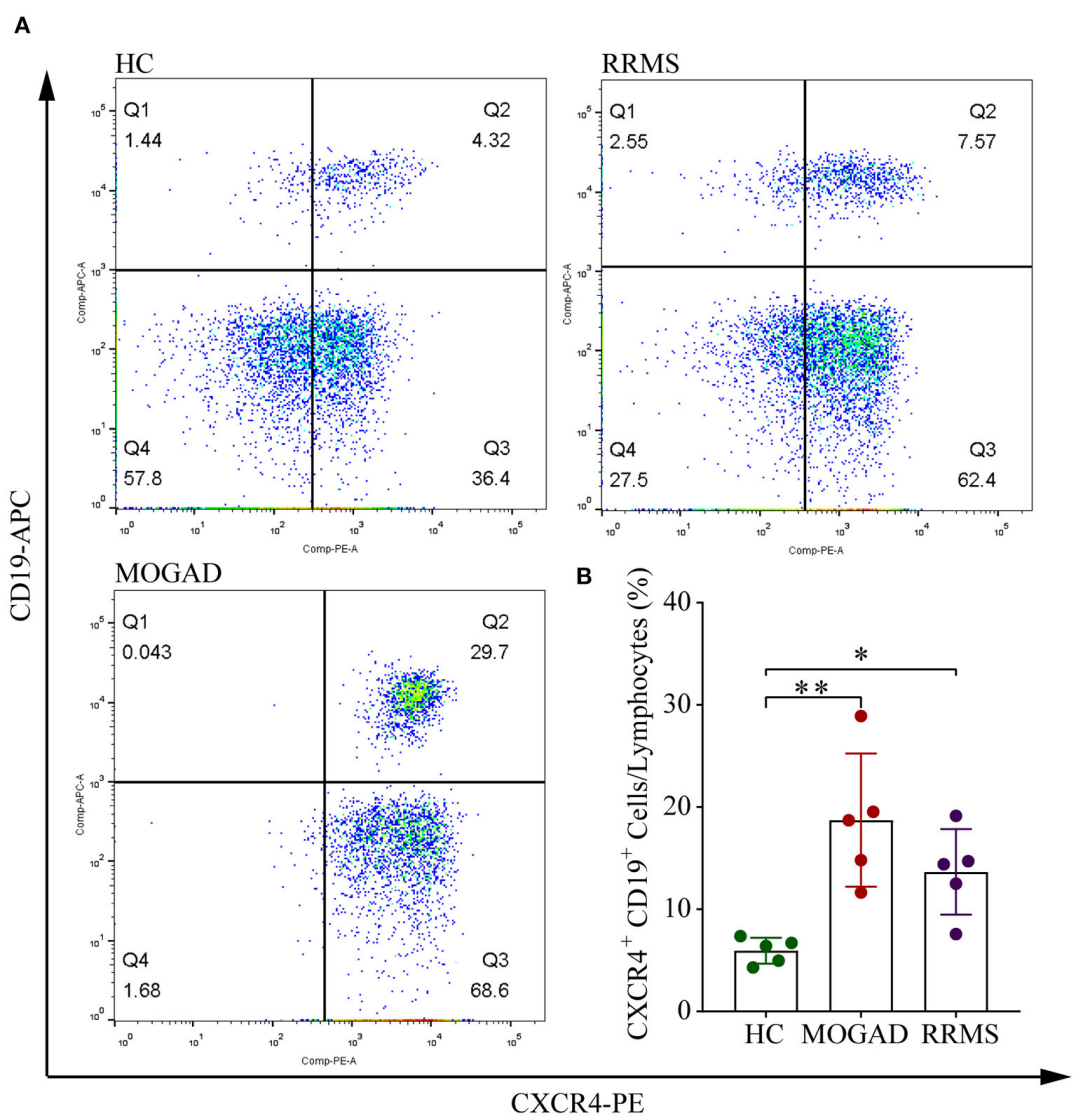
Flow cytometry analysis of CD19^+^ CXCR4^+^ B cell subsets in HC, RRMS, and MOGAD. **(A)** Gating strategy of CD19^+^ CXCR4^+^ by flow cytometry. **(B)** Percentages of CD19^+^ CXCR4^+^ B cells of HC, MOGAD, and RRMS. HC, healthy controls; RRMS, relapsing-remitting multiple sclerosis; MOGAD, myelin oligodendrocyte glycoprotein (MOG) antibody-associated disease; PBMC, peripheral blood mononuclear cell. The data represent the mean ± SEM. *p < 0.05, **p < 0.001.

Among four B cell subsets, the naive B cells (B2) were increased in RRMS and MOGAD by ~4.29 and 5.00%, respectively, compared to HCs (~2.60%; Figure 4B and Supplementary Table 2). The increase of naive B cells in these two diseases was confirmed by flow cytometry, which was consistent with single-cell sequencing data (Figure 5 and Supplementary Figure 2). In addition, a comparison between RRMS and MOGAD revealed that the fraction of plasma cells (B1) and memory B cells (B3) numerically was reduced in MOGAD. In summary, four different subsets were identified in B cells and further revealed an expansion of naive B cells in both RRMS and MOGAD. The results also showed a reduction in the fraction of plasma and memory B cells in MOGAD than in RRMS.

## Discussion

This study aimed to comprehensively identify the circulating immune-cell subset properties of peripheral blood in the patients with RRMS and MOGAD using scRNA-seq. The results showed the distinct immune cell signatures in RRMS and MOGAD.

Previous studies have shown that myeloid cells play protective roles in neuroinflammation (28–30). In this study, based on the established marker database, four distinct myeloid cell subsets were identified: classical (CD14^++^ CD16^−^), non-classical (CD14^+^ CD16^++^), and intermediate (CD14^++^ CD16^+^) monocytes. The CD16^+^ monocytes play key immune surveillance roles in CNS by shifting to the inflammation sites and breaking the blood-brain barrier in neuroinflammation (31). The fraction of intermediate (CD14^++^ CD16^+^) monocytes was observed to increase in patients with RRMS and MOGAD than in HCs, suggesting that targeting CD16^+^ monocytes may be a feasible therapeutic strategy for RRMS and MOGAD (32), a finding which is consistent with reports from other studies (33–35). The inflammatory monocytes (M2), specifically enriched in MOGAD, expressed a high level of S100A8, S100A9, and S100A12 in the serum of patients with diverse inflammatory diseases (36, 37). The results above suggest that these inflammatory monocytes may serve as potential diagnostic indicators for MOGAD.

Inflammatory T cells play critical roles in the pathogenesis of neuroinflammatory autoimmune diseases (38, 39). Studies show that CD4^+^ and CD8^+^ T cells are associated with demyelinating lesions and axonal damage (40, 41). In this study, a detailed analysis of the T cells identified 2 CD4^+^ and 6 CD8^+^ T cell subsets. Previous studies showed that the elevated cytotoxic CD8^+^ T cells play central roles in MS development by recognizing myelin basic protein (42). Similarly, increased fractions of cytotoxic CD8^+^ T cells were present in both RRMS and MOGAD, illustrating that these cells may be an indicator of the disease progression. Cytotoxic NK cells participate in the regulation of immune response and contribute to the pathogenesis of numerous autoimmune diseases (43–46). In this study, the sub-cluster analysis identified the cytotoxic memory-like NK cells, which were markedly expanded in the two diseases, especially RRMS, suggesting that the cells may contribute to disease pathogenesis.

The B cells are important weapons against infectious diseases and also contribute to numerous autoimmune diseases, such as MS (16). Evidence proves that target depletion of CD20^+^ B cells can effectively suppress inflammatory activities in MS (47–49). After anti-CD20 treatment, the patients with MS demonstrated a reconstituted B cell repertoire different from those not receiving treatment. The reconstituted B cells were naive and can produce less proinflammatory cytokine; for example, an increase in interleukin-10 (IL-10) level may be attributed to the decrease in proinflammatory responses of T cell and myeloid-lineage across the reconstitution phase (50–52). B cells can also weaken the immune response of different stages of CNS inflammation by secreting a set of anti-inflammatory cytokines, namely, IL-10, transforming growth factor beta (TGF-β), and IL-35) (53). In this study, the relative abundance of naive B cells was increased in RRMS and MOGAD, coupled with high levels of IL-10 (54, 55). These results suggest that targeted depletion of B cells could be a feasible strategy on RRMS and MOGAD.

Our findings indicate that the two diseases exhibit distinct immune cell signatures, but several limitations should be mentioned in this study. Due to the fact that some patients were unwilling to take part in this research, the number of subjects was relatively small, which would make the result heavily depend on the specific characteristics of these individuals, such as disease stage, previous infections, other immune-related disorders/conditions, and genetic factors. Last but not least, less-frequent immune-cell populations were not identified and characterized using scRNA-seq in this work, which needs to be further studied.

## Conclusion

In this study, we described unique peripheral blood single-cell transcriptome profiles in RRMS and MOGAD. RRMS was characterized by increased naive CD8^+^ T cells and cytotoxic memory-like NK cells, together with decreased inflammatory monocytes, whereas MOGAD exhibited increased inflammatory monocytes and cytotoxic CD8 effector T cells, coupled with decreased plasma cells and memory B cells. These findings allow for highly predictive discrimination of these two diseases and pave a novel avenue for the diagnosis and therapy of neuroinflammatory diseases.

## Data Availability Statement

The datasets presented in this study can be found in online repositories. The names of the repository/repositories and accession number(s) can be found in the article/Supplementary Material.

## Ethics Statement

The study protocols were approved by the Ethics Committee Board of the First Affiliated Hospital of Zhengzhou University (2021-KY-0588-002). The patients/participants provided their written informed consent to participate in this study.

## Author Contributions

HL and JL designed the research, contributed valuable advice, and edited the manuscript. JP and XY conducted the research and collected data. ZW and MC analyzed the data and performed the statistical analysis. JL wrote the draft of the main manuscript. All authors drafted the manuscript, performed the revision, and approved the final version of the manuscript.

## Funding

This work was supported by the Medical Science and Technology Research Project of Henan Province (no. LHGJ20190086) and the National Natural Science Foundation of China (no. U2004128).

## Conflict of Interest

The authors declare that the research was conducted in the absence of any commercial or financial relationships that could be construed as a potential conflict of interest.

## Publisher's Note

All claims expressed in this article are solely those of the authors and do not necessarily represent those of their affiliated organizations, or those of the publisher, the editors and the reviewers. Any product that may be evaluated in this article, or claim that may be made by its manufacturer, is not guaranteed or endorsed by the publisher.

## Supplementary Material

Supplementary Figure 1 Consistency of identification and cell capture in PBMC scRNA-seq. PBMC, peripheral blood mononuclear cells.

Supplementary Figure 2 Gating strategy of CD19^+^CXCR4^+^ by flow cytometry.
