# Supplementary figures and images for: Single-Cell Transcriptome Profiling Unravels Distinct Peripheral Blood Immune Cell Signatures of RRMS and MOG Antibody-Associated Disease
Source: Front Neurol. 2022 Jan 14;12:807646. doi: 10.3389/fneur.2021.807646 (PMC8795627; doi:10.3389/fneur.2021.807646)

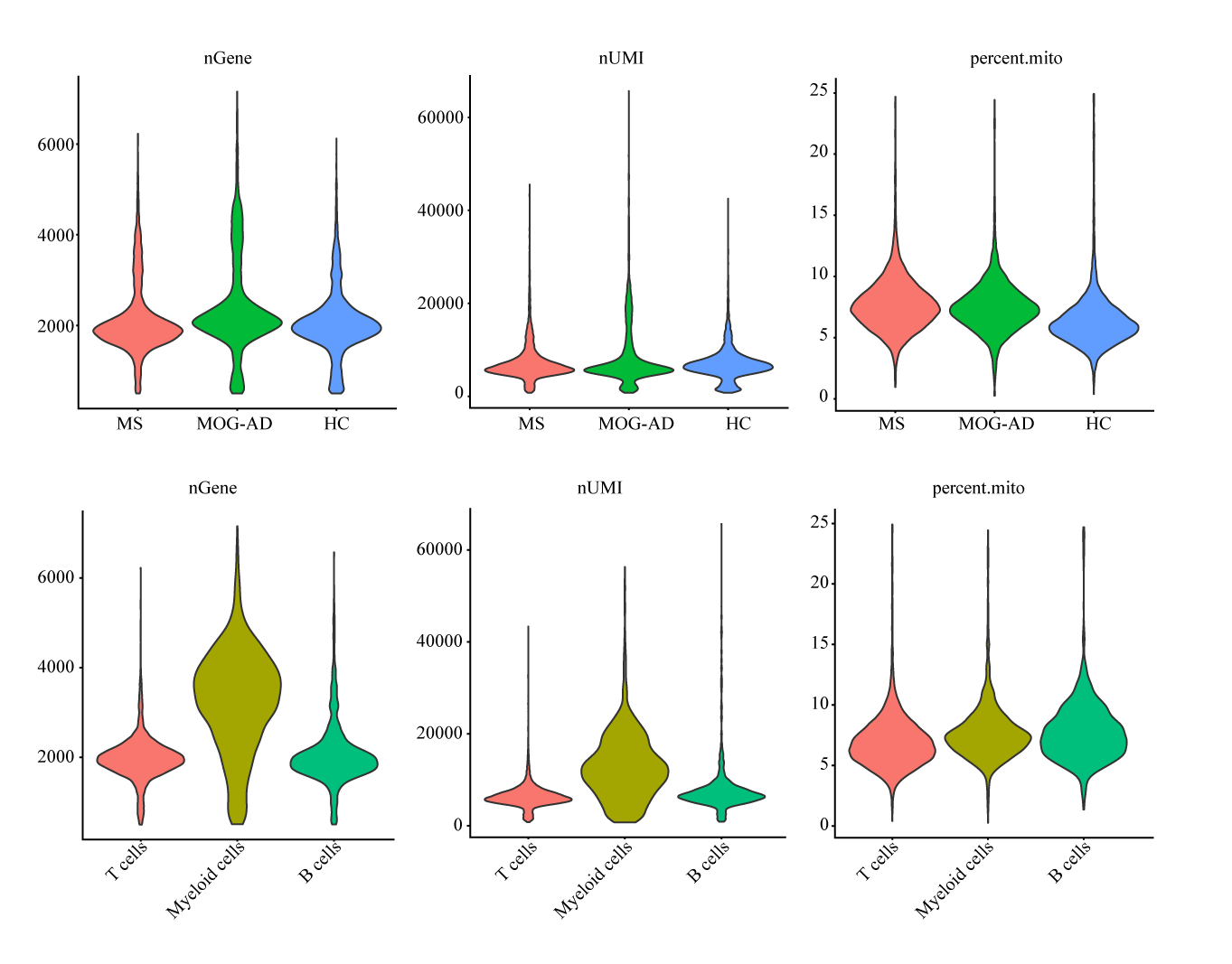

Supplement: Supplementary Figure 1 — Consistency of identification and cell capture in PBMC scRNA-seq. PBMC, peripheral blood mononuclear cells. [file Image_1.TIF]

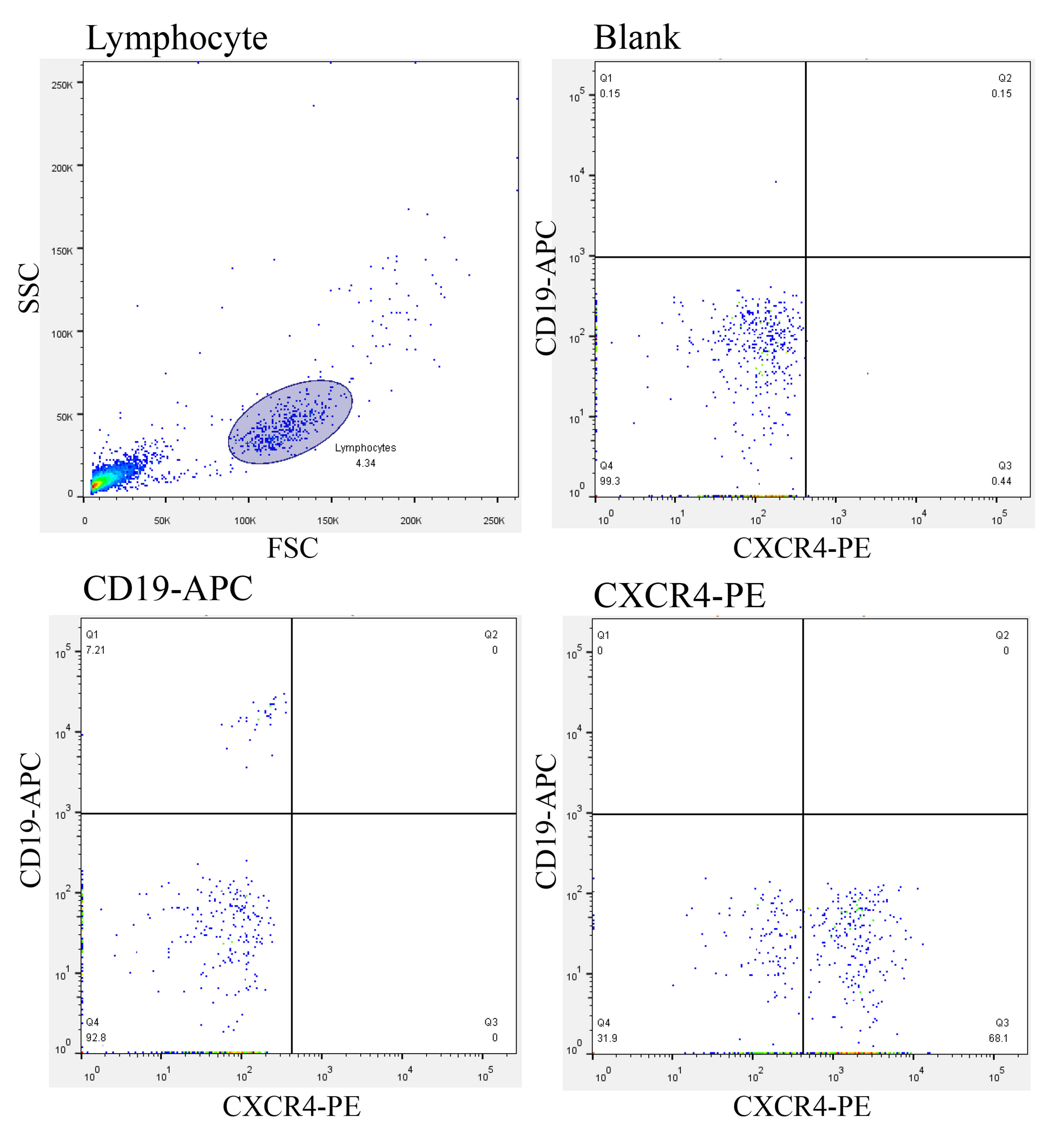

Supplement: Supplementary Figure 2 — Gating strategy of CD19+CXCR4+ by flow cytometry. [file Image_2.TIF]
